# 伴第11号染色体长臂异常的Burkitt样淋巴瘤二例报告并文献复习

**DOI:** 10.3760/cma.j.issn.0253-2727.2021.12.013

**Published:** 2021-12

**Authors:** 奕 夏, 震 王, 莉 王, 浩睿 申, 华 尹, 佳竹 吴, 卫 徐

**Affiliations:** 1 南京医科大学第一附属医院，江苏省人民医院血液科，南京医科大学血液研究重点实验室，江苏省肿瘤个体化医学协同创新中心 210029 Department of Hematology, the First Affiliated Hospital of Nanjing Medical University, Jiangsu Province Hospital, Key Laboratory of Hematology of Nanjing Medical University, Collaborative Innovation Center for Cancer Personalized Medicine, Nanjing 210029, China; 2 南京医科大学第一附属医院，江苏省人民医院病理科 210029 Department of Pathology, the First Affiliated Hospital of Nanjing Medical University, Jiangsu Province Hospital, Nanjing 210029, China

伴第11号染色体长臂异常的Burkitt样淋巴瘤（Burkitt-like lymphoma with 11q aberration, BLL-11q）是2016年WHO淋巴组织病变分类修订版中新提出的一个暂定亚型[1]。此类淋巴瘤具有生发中心表型，尽管具有与Burkitt淋巴瘤（BL）相似的形态学和免疫表型特征，却缺乏MYC基因重排，而代之以特征性的第11号染色体长臂近端拷贝数扩增和端粒区丢失，最小拷贝数增加区域为11q23.2-23.3，最小拷贝数缺失区域为11q端粒端11q24.1。此类淋巴瘤罕见，与BL相比，染色体核型更为复杂，基因突变谱也大相径庭[1]–[5]。目前BLL-11q应归于BL、弥漫大B细胞淋巴瘤（DLBCL）还是高级别B细胞淋巴瘤（HGBCL）尚无定论。现报道本院2021年1月和3月收治的2例患者，并进行文献复习。

## 病例资料

例1，男，22岁，因“发现颈部左侧肿物2个月”入院，否认发热、盗汗、体重减轻等全身症状。无特殊既往史和家族史。体格检查：美国东部肿瘤协作组体能状态（ECOG-PS）评分0分，颈部左侧触及一肿大淋巴结，直径2 cm，质硬，无压痛。血常规、乳酸脱氢酶、β_2_微球蛋白、肝肾功能、EBV-DNA、HBV五项、HIV抗体、骨髓常规、骨髓活检、免疫球蛋白重排、流式细胞术检查及脑脊液常规、生化、形态学检查等均未见异常。全身PET-CT显示：颈部左侧颌下一直径约2.5 cm肿大淋巴结影，SUVmax 25.1。颈部左侧淋巴结穿刺活检，HE染色低倍镜下见弥漫性淋巴样病变细胞增生浸润，淋巴结结构破坏；高倍镜下病变细胞中等大小，单一形态，星空现象，且伴有较多的核碎片簇状聚集；免疫组化：肿瘤细胞CD20（+）、PAX-5（+），表达生发中心标志CD10、BCL6，同时CD38（+）、p53（60％+）、C-MYC（40％+）、Ki-67（90％+）。MUM1、BCL2、LMO2、原位杂交EBER均为阴性。荧光原位杂交（FISH）：采用IGH/BCL2融合探针、BCL6分裂探针、C-MYC分裂探针、p53探针未见异常；采用11q23.3/11q24.3探针：镜下见部分肿瘤细胞内显示两红一绿或三红一绿信号，所占比例约30％，提示11q24.3缺失，故诊断为BLL-11q（图1）。燃石利清靶向测序芯片（广州燃石医学检验所有限公司产品）测得该患者血清中突变基因包括：KMT2D p.G5001E（VAF 36.06％）、EP300 p.S1977G（VAF 8.20％）和TET2 p.I1873T（VAF 11.08％）。采用利妥昔单抗联合Hyper CVAD方案（环磷酰胺、长春地辛、脂质体多柔比星、地塞米松）联合鞘内注射3个疗程后达完全缓解（CR），目前持续治疗中。

**图1:**
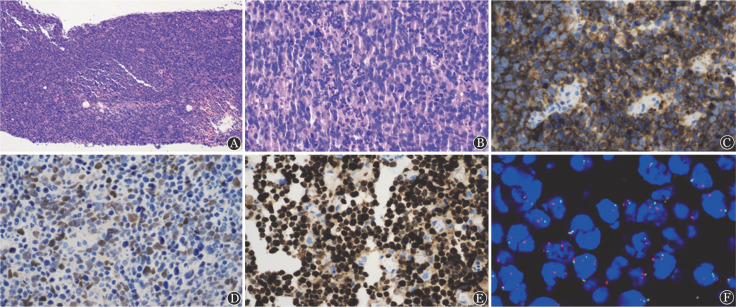
例1肿瘤细胞病理特征 A：弥漫性淋巴样病变细胞增生浸润，淋巴结结构破坏（HE染色，×100）；B：病变细胞中等大小，单一形态，星空现象，且伴有较多的核碎片簇状聚集（HE染色，×400）；C：肿瘤细胞CD10染色阳性（×400）；D：C-MYC染色40％阳性（×400）；E：Ki-67染色90％阳性（×400）；F：采用11q23.3/11q24.3探针，镜下见部分肿瘤细胞内为两红一绿或三红一绿信号，所占比例约30％

例2，男，14岁，因“双侧鼻塞、涕中带血5个月余”入院，否认发热、盗汗、体重减轻等全身症状。无特殊既往史和家族史。体格检查：ECOG-PS评分0分，鼻黏膜充血，鼻中隔稍偏曲，浅表未及肿大淋巴结。血常规、乳酸脱氢酶、β_2_微球蛋白、肝肾功能、EBV-DNA、HBV五项、HIV抗体、骨髓常规、骨髓活检、免疫球蛋白重排、流式细胞术检查及脑脊液常规、生化、形态学检查等均未见异常。全身PET-CT显示：鼻咽部5.7 cm×3.1 cm软组织肿物，密度均匀，SUVmax 17.3。鼻咽部肿物切除活检，HE染色病变形态有一定异质性，部分区病变细胞呈中等大小，核碎片散在聚集；部分区病变细胞呈DLBCL形态，星空现象明显；部分区病变细胞完全呈DLBCL形态。免疫组化：肿瘤细胞CD20（+）、PAX-5（+），表达生发中心标志CD10和BCL6，同时C-MYC（>40％+）、CD38（弱+）、Ki-67（>85％+）。MUM1、BCL2、LMO2、原位杂交EBER均为阴性。FISH：采用IGH/BCL2融合探针、BCL6分裂探针、C-MYC分裂探针、p53探针未见异常；采用11q23.3/11q24.3探针：荧光显微镜下见染色体11q23.3呈多个红色信号的比例>50％，染色体11q24.3呈单个绿色信号的比例>50％，提示染色体11q23.3的拷贝数扩增，染色体11q24.3的拷贝数缺失（图2）。燃石利清靶向测序芯片测得该患者肿瘤组织中突变基因包括：PTEN p.Y46S（VAF 28.28％），CCND1 cn_amp（VAF 3.3％），GNA13 p.F325V（VAF 24.36％），GNA13 c.561+2T>G（VAF 33.49％），PIK3CA p.V344G（VAF 26.02％），DDX3X p.F357fs（VAF 50.15％）。采用利妥昔单抗联合Hyper CVAD方案联合鞘内注射3个疗程后达CR，目前持续治疗中。

**图2:**
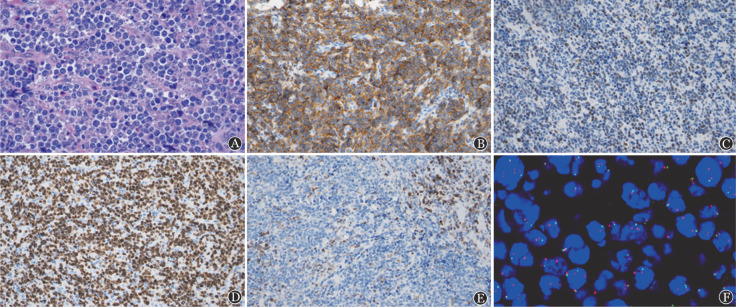
例2肿瘤细胞病理特征 A：肿瘤细胞形态有异质性，部分区病变细胞呈中等大小，核碎片散在聚集；部分区病变细胞呈DLBCL形态，星空现象明显；部分区病变细胞完全呈DLBCL形态（HE染色，×400）；B：肿瘤细胞CD10染色阳性（×200）；C：C-MYC染色>40％阳性（×200）；D：Ki-67染色90％阳性（×200）；E：BCL2染色阴性（×200）；F：采用11q23.3/11q24.3探针，镜下见11q23.3呈多个红色信号的比例>50％，11q24.3呈单个绿色信号的比例>50％

## 讨论及文献复习

BLL-11q罕见，既往报道中占BL患者不足10％[2]。Gonzalez-Farre等[4]在95例既往诊断为BL、不典型BL或高级别B细胞淋巴瘤，非特指型（HGBCL-NOS）的患者中筛查，11q异常发生率为8％，均出现于<40岁的青年患者中。Horn等[5]对35例MYC重排阴性的既往诊断为BL、DLBCL-BL或HGBCL-NOS的患者进行11q异常FISH检测，发现11q异常阳性者占52％，其中MYC重排阴性的BL中75％（12/16）存在11q异常，MYC重排阴性的HGBCL中21％（3/14）存在11q异常，而在MYC重排阴性的既往诊断为DLBCL的患者中，仅2％（1/62）存在11q异常。但值得一提的是，仍有极少数MYC重排阳性的侵袭性淋巴瘤伴随11q异常[5]–[6]。

BLL-11q男性多见，好发于儿童及青少年，中位发病年龄在15～20岁[1]–[4]，但也有高至82岁的BLL-11q的病例报道[7]。BLL-11q病因不明，与EBV感染无关，有报道BLL-11q在器官移植后免疫缺陷的患者中常见，占移植后BL的43％（3/7），且均为EBV阴性（3/5）[8]。BLL-11q以单个淋巴结起病为主，最常见累及的部位包括头颈部淋巴结、腹腔淋巴结、腹股沟淋巴结等，少数病例也有结外器官（肠道、阑尾、骨）累及[2]–[4]。绝大部分BLL-11q病灶局限，Gonzalez-Farre等[4]报道的11例患者中，仅3例分期为Ⅲ～Ⅳ期。

病理形态上，BLL-11q与BL相似，肿瘤细胞弥漫性生长，中等大小，单一形态，核圆、核仁小，胞质嗜碱，吞噬细胞“星空现象”多见[1]–[4]。但与BL相比，BLL-11q核碎片现象更为明显，在一个包含33例患者的双盲研究中，“核碎片”现象预测潜在的11q异常的特异性达到91％[5]。

BLL-11q呈现与BL相似的生发中心免疫表型，表达CD10、BCL6，不表达BCL2、MUM1，Ki-67指数大于90％。但BLL-11q EBV均为阴性，C-MYC蛋白水平显著低于BL，仅呈局灶弱阳性。40％～70％的BLL-11q表达生发中心标志LMO2，而LMO2表达罕见于BL。此外，少数BLL-11q患者表达CD56，Rymkiewicz等[9]利用流式细胞术发现CD16/CD56阳性仅存在于BLL-11q中（BLL-11q 60％，BL 0％），并提出CD16/CD56阳性不伴CD38高表达是鉴别CD10^+^侵袭性淋巴瘤中BLL-11q的有效手段。

细胞遗传学特征方面，BLL-11q拷贝数变异（CNA）多，中位CNA数6.5～7.1，与BL相似，但复杂核型更常见。BLL-11q缺乏MYC重排，也没有BCL2和BCL6重排，典型的细胞遗传学改变为11q23拷贝数增加合并11q端粒端11q24.1缺失。但并非所有BLL-11q均具有这两种异常。Wagener等[3]和Gonzalez-Farre等[4]报道的26例BLL-11q中，20例呈现典型的11q23拷贝数增加合并11q端粒端11q24.1缺失；4例仅有11q端粒端11q24.1缺失，无11q23拷贝数增加；1例为复杂11q改变；1例呈现11q23.3-q25拷贝数中性的杂合性缺失。其他常见的细胞遗传学异常包括12号染色体三体（最小拷贝数增加区域12q13.11-q24.32）、6q12.1-q21缺失、7q端粒端7q.34拷贝数增加、13q32.3-q34缺失、5q21.3-q32拷贝数增加等。

BLL-11q与BL的基因突变谱显著不同。对15例BLL-11q患者标本进行全外显子测序显示，此类淋巴瘤最常见的突变基因包括GNA13（47％）、TTN（47％）、FAT4（40％）、PKD1L2（33％）、NFRKB（27％）、DDX3X（27％）、TENM3（27％）等[3]；另一项针对11例BLL-11q患者标本的靶向测序结果则提示BTG2（40％）、ETS1（30％）、EP300（30％）、DDX3X（30％）、GNA13（30％）为常见突变基因。除了GNA13和DDX3X之外，BLL-11q突变谱缺乏BL中常见的突变基因如MYC、ID3、TCF3、TP53、SMARCA4、CCND3等，却与GCB来源的其他淋巴瘤存在部分重叠，例如常出现BTG2、DDX3X、ETS1、EP300、GNA13及表观遗传学调控基因EP300、KMT2D、EZH2等异常[4]。

尽管BLL-11q基因异常众多，但其发病机制仍不清楚。研究者进一步探索了是否存在位于11q最小扩增区域或最小缺失区域的驱动基因。2014年Salaverria等[2]对16例BLL-11q患者进行突变检测，发现4例BLL-11q患者存在位于最小缺失区域11q24.1-ter的ETS1基因的点突变。ETS1是转录因子，参与众多基因转录调控，影响干细胞发育、细胞衰老和死亡以及肿瘤的发生。2019年Wagener等[3]对15例BLL-11q进行全外显子测序，也发现2例患者具有ETS1突变，此外他们还发现4例患者存在同样位于最小缺失区域11q24.1-ter的NFRKB基因突变。此4例中3例为无义突变，导致NFRKB基因转录提前终止。比较既往基因表达谱数据也证实，BLL-11q患者NFRKB基因表达水平显著低于BL患者。NFRKB是INO80染色质重塑复合物，在转录调控中发挥作用。分析INO80染色质重塑复合物中的所有基因发现，15例BLL-11q患者中有5例存在相关基因突变。ChIP-seq数据库提示NFRKB可结合位于最小拷贝数增加区域11q23.2-23.3的5个过表达基因（BLL-11q对BL）IL10RA、ZNF259、PAFAH1B2、CEP164、SIDT2的转录起始位点，但不能与位于最小缺失区域11q24.1-ter的任何基因结合，提示NFRKB基因缺失可能通过改变核小体构象、提高基因转录水平等导致BLL-11q出现最小扩增区域，而位于INO80染色质重塑复合物的基因突变也可能是导致BLL-11q发病的驱动基因。

BLL-11q罕见，缺乏规范的治疗方案，目前大多数中心仍倾向于采取BL样治疗。Sevilla等[10]报道对MYC重排阴性的HGBCL采用R-CHOP样治疗预后显著差于采用更高化疗强度（如BL样方案）的MYC重排阳性的HGBCL患者。Salaverria等[2]则发现，同样采用BL样治疗，BLL-11q预后优于BL。后续报道采用BL样方案，几乎所有患者均可获得CR，5年总生存率也可达到80％以上[4],[9],[11]。

本报道中2例患者均为青少年，病灶局限，肿瘤细胞具有典型的病理形态、生发中心样免疫表型和特征性的11q染色体异常，靶向测序存在GNA13、DDX3X、KMT2D、EP300等BLL-11q常见基因突变，经利妥昔单抗联合HyperCVAD方案治疗后中期评估均获得CR，符合BLL-11q特征。此类淋巴瘤目前虽被称为Burkitt样淋巴瘤，但越来越多的证据表明它与BL及其他类型的侵袭性B细胞淋巴瘤均存在遗传学上的本质差异，是一种独立类型的淋巴瘤。临床上，对于形态学具有BL或BL/DLBCL特征且呈生发中心样免疫表型、Ki-67指数高的患者，如无MYC重排，需加做11q23.3/11q24.3 FISH探针检查，排查BLL-11q的可能性，提高诊断准确率。
